# Supplementary figures and images for: Elevated DNA damage without signs of aging in the short-sleeping Mexican cavefish
Source: eLife. 2025 Nov 14;13:RP99191. doi: 10.7554/eLife.99191 (PMC12618005; doi:10.7554/eLife.99191)

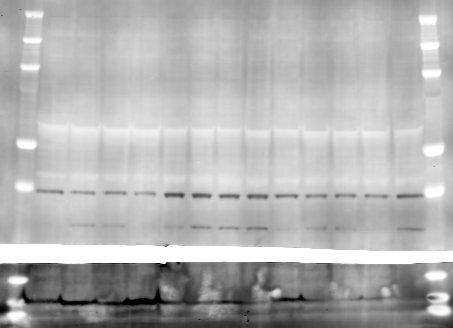

Supplement: Figure 4—source data 1. [file elife-99191-fig4-data1.zip › Figure 4 - source data 1/C/0000020_01_800-gel1.jpg]

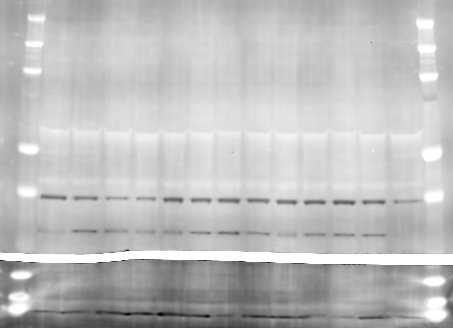

Supplement: Figure 4—source data 1. [file elife-99191-fig4-data1.zip › Figure 4 - source data 1/C/0000020_01_800-gel2.jpg]

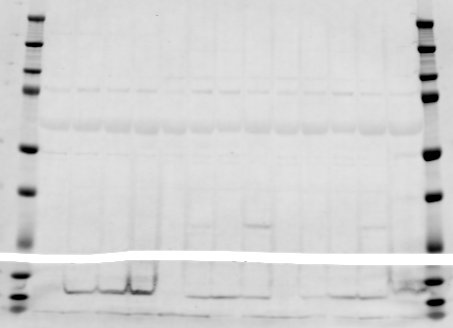

Supplement: Figure 4—source data 1. [file elife-99191-fig4-data1.zip › Figure 4 - source data 1/C/0000020_01_700-gel2.jpg]

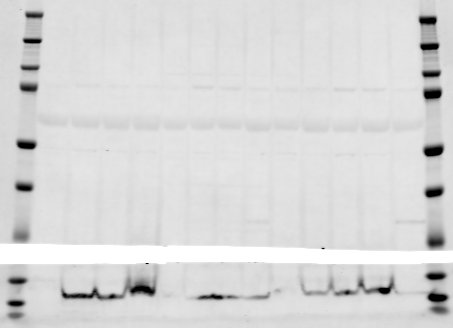

Supplement: Figure 4—source data 1. [file elife-99191-fig4-data1.zip › Figure 4 - source data 1/C/0000020_01_700-gel1.jpg]

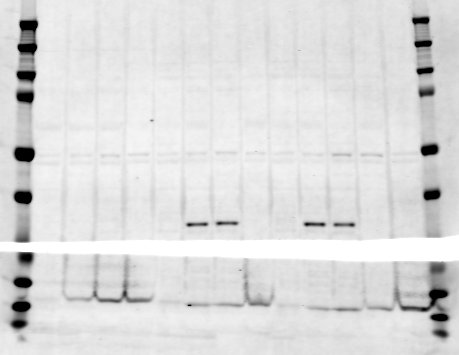

Supplement: Figure 4—figure supplement 1—source data 1. [file elife-99191-fig4-figsupp1-data1.zip › Supplemental Figure 4 - source data 1/D/0000022_01_700-gel2.jpg]

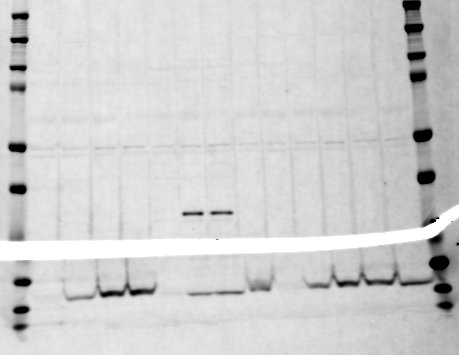

Supplement: Figure 4—figure supplement 1—source data 1. [file elife-99191-fig4-figsupp1-data1.zip › Supplemental Figure 4 - source data 1/D/0000022_01_700-gel1.jpg]

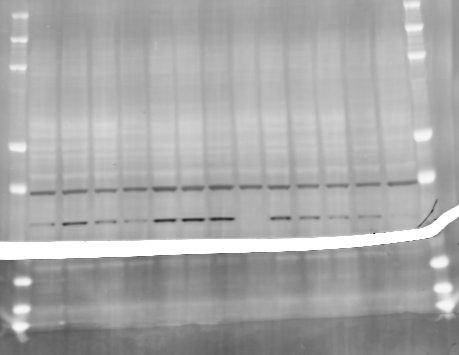

Supplement: Figure 4—figure supplement 1—source data 1. [file elife-99191-fig4-figsupp1-data1.zip › Supplemental Figure 4 - source data 1/D/0000022_01_800-gel1.jpg]

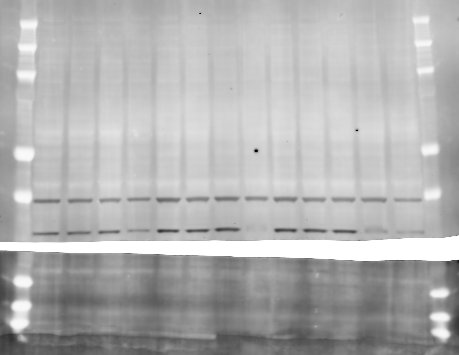

Supplement: Figure 4—figure supplement 1—source data 1. [file elife-99191-fig4-figsupp1-data1.zip › Supplemental Figure 4 - source data 1/D/0000022_01_800-gel2.jpg]
