# Supplementary material for: Elevated DNA damage without signs of aging in the short-sleeping Mexican cavefish
Source: eLife. 2025 Nov 14;13:RP99191. doi: 10.7554/eLife.99191 (PMC12618005; doi:10.7554/eLife.99191)
Supplement: Figure 4—source data 2. [file elife-99191-fig4-data2.zip › Figure 4 - source data 2/Figure 4C -sourse data 2.pptx]

## Slide 1
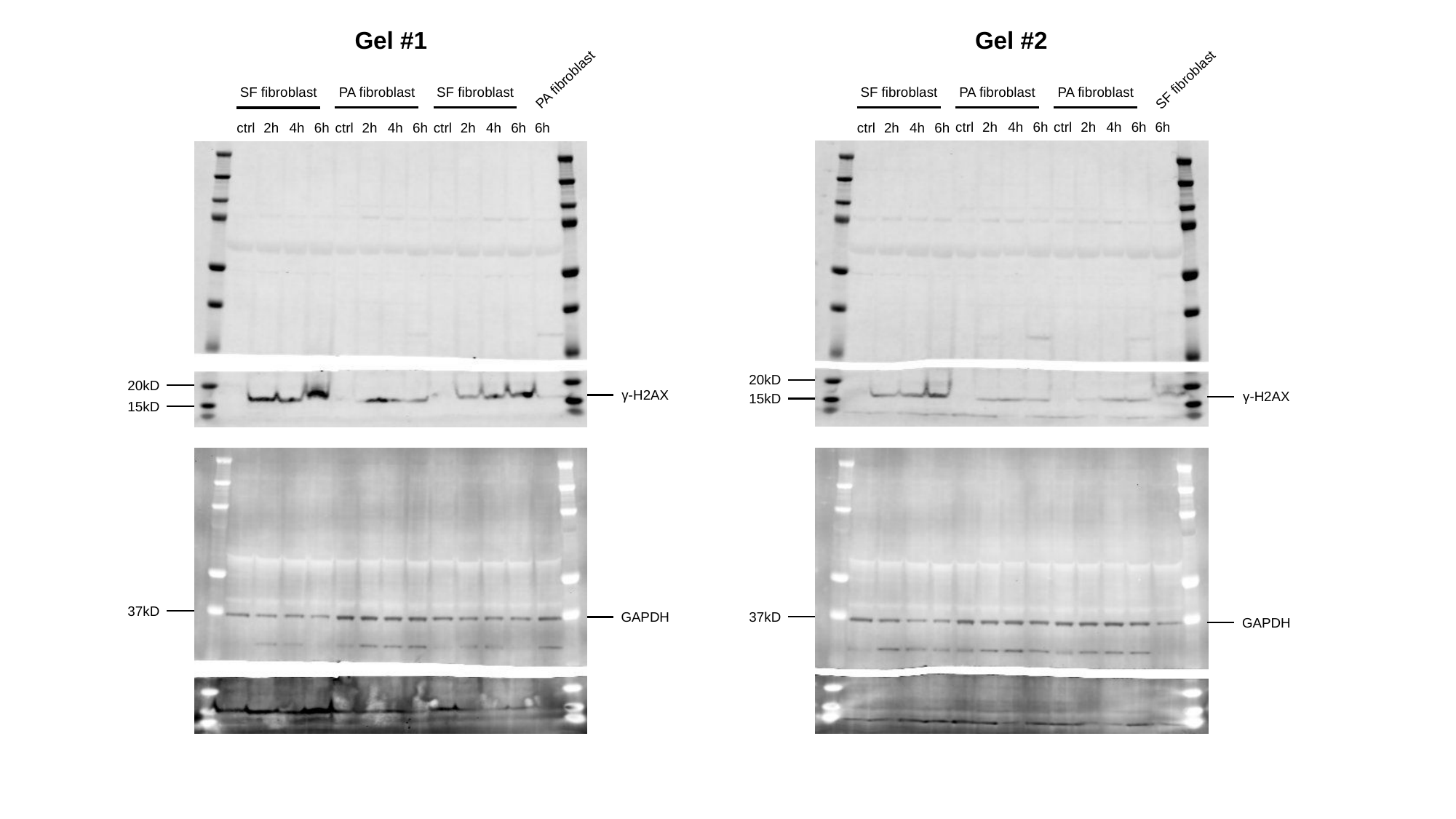

Gel #1
Gel #2
SF fibroblast
PA fibroblast
PA fibroblast
PA fibroblast
PA fibroblast
SF fibroblast
SF fibroblast
SF fibroblast
ctrl
2h
4h
6h
ctrl
2h
4h
6h
6h
ctrl
2h
4h
6h
ctrl
2h
4h
6h
ctrl
2h
4h
6h
6h
ctrl
2h
4h
6h
20kD
20kD
γ-H2AX
γ-H2AX
15kD
15kD
37kD
37kD
GAPDH
GAPDH
