# Supplementary material for: Elevated DNA damage without signs of aging in the short-sleeping Mexican cavefish
Source: eLife. 2025 Nov 14;13:RP99191. doi: 10.7554/eLife.99191 (PMC12618005; doi:10.7554/eLife.99191)
Supplement: Figure 4—figure supplement 1—source data 2. [file elife-99191-fig4-figsupp1-data2.zip › Supplemental Figure 4 - source data 2/Supplemental Figure 4D -sourse data 2.pptx]

## Slide 1
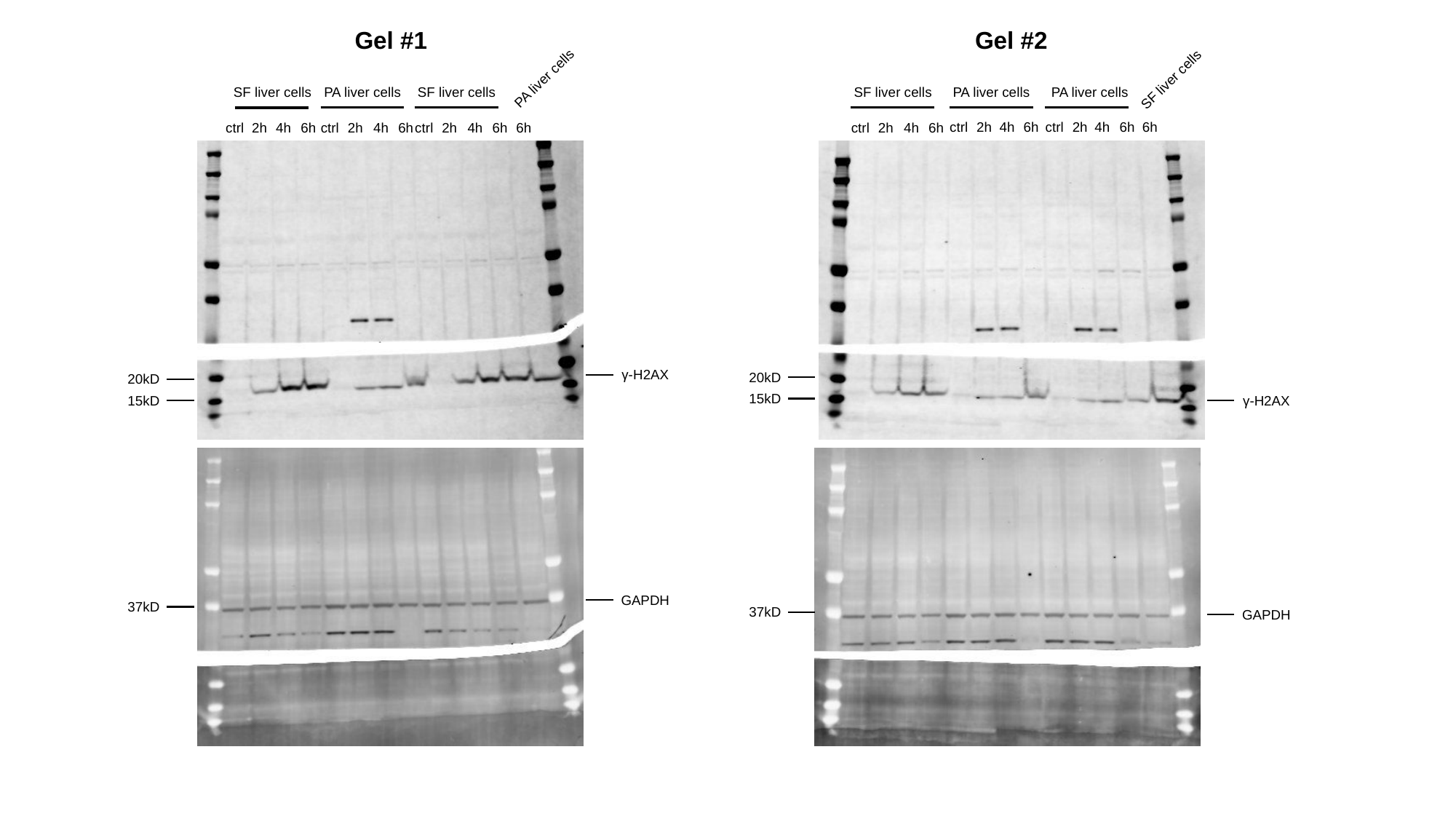

Gel #1
Gel #2
PA liver cells
SF liver cells
PA liver cells
PA liver cells
SF liver cells
PA liver cells
SF liver cells
SF liver cells
ctrl
2h
4h
6h
ctrl
2h
4h
6h
6h
ctrl
2h
4h
6h
ctrl
2h
4h
6h
ctrl
2h
4h
6h
6h
ctrl
2h
4h
6h
γ-H2AX
20kD
20kD
15kD
15kD
γ-H2AX
GAPDH
37kD
37kD
GAPDH
